# Transoral Resection of Giant Parapharyngeal Space Tumors via a Combined Surgical Approach

**Published:** 2019-03

**Authors:** Konstantinos Markou, Sarantis Blioskas, Argyrios Krommydas, George Psillas, Petros Karkos

**Affiliations:** *Department of Otorhinolaryngology - Head and Neck Surgery, Aristotle University of Thessaloniki, AHEPA Hospital, 1 Stilponos Kyriakidi St, 54636 Thessaloniki, Greece.*

**Keywords:** Mandibulotomy, Prestyloid parapharyngeal space, Transoral

## Abstract

**Introduction:**

Parapharyngeal space (PPS) tumors account for 0.5% of the head and neck neoplasms. Based on the evidence, 80% of these tumors are of a benign nature. Surgical excision is the treatment of choice for this condition. The present study was conducted to propose transoral resection as an efficient way to excise the benign well-defined tumors of the PPS.

**Materials and Methods:**

This retrospective case series study was conducted on seven patients undergoing the transoral excision of the sizeable masses of the PPS via a combined approach. Computed tomography and magnetic resonance scans revealed giant masses in the PPS in all cases. These neoplasms were preoperatively diagnosed as well-delineated, non-vascular, and benign.

**Results:**

All patients underwent transoral tumor excision preceded by an auxiliary transcervical approach, which served as an assurance for the dissection and preservation of the cranial nerves and neurovascular bundle without any tumor spillage. Average hospital stay was limited to a maximum of 3 days, and all patients had an uneventful postoperative course. The follow-up examination did not indicate any recurrence.

**Conclusion:**

Based on the findings, transoral resection can be concluded as an efficient way to excise benign, well-defined tumors of the PPS. This procedure appears to be safe when a secondary transcervical approach is applied. Given the unnecessity of performing mandibulotomy in this procedure, it is expected to have lower morbidity and fewer complications.

## Introduction

Tumors of the parapharyngeal space (PPS) account for only 0.5% of the head and neck neoplasms (1-2). They are mostly diagnosed in adults; however, they can be rarely found in pediatric population (3). A wide spectrum of neoplasms of different histological types arises in the compartments of this space. Salivary gland tumors, most commonly pleomorphic adenomas (4), have the highest occurrence, followed by paragangliomas or neurogenic tumors. 

About 20% of PPS neoplasms are malignant, whereas 80% of them are benign (5). Imaging modalities are of crucial importance in order to accurately and efficiently assess each individual case. As a general rule, prestyloid lesions arise from the salivary gland tumors, which displace the internal carotid artery (ICA) posteriorly, while the ICA is anteromedially displaced by poststyloid space lesions (6). 

As surgical excision is the mainstay of treatment, a variety of surgical procedures have been utilized in the management of these lesions. However, transoral approach remains to this day reserved for specific cases, as it is generally considered unsafe. Consequently, sizeable lesions are mainly dealt via a transcervical or transparotid approach, usually necessitating an auxiliary mandibulotomy in order to gain wider exposure.

Herein, we presented a series of seven patients bearing sizeable benign lesions of the prestyloid PPS. They all underwent transoral tumor excision, preceded by the implementation of a main transoral method and a secondary transcervical approach. The use of a combined approach, though eliminating the need to perform a mandibulotomy, provides satisfactory access and adequately secures the neurovascular bundle and lower cranial nerves. 

## Materials and Methods 

This case series was conducted on seven patients (i.e., 4 females and 3 males) bearing sizeable PPS tumors during the last 6 years. All patients were referred to our department due to the vast dimension of the lesion and/or the inherent difficulty of the surgical exploration of PPS. The age of the patients varied from 32 to 72 years. Symptomatology included a vast diversity of non-specific symptoms, such as sleep apnea or foreign body sensation. It is noteworthy that two cases (i.e., patients 6 and 7) were asymptomatic. The lesions found suspected during the random clinical examination or discovered through incidental imaging, undertaken due to unrelated causes. In all cases, physical examination revealed the considerable bulging of the soft palate, occasionally resulting in the concurrent anterolateral dislocation of the ipsilateral tonsil. All patients underwent computed tomography (CT) scan that indicated a sizeable PPS tumor extending roughly from the skull base to the ipsilateral submandibular gland depending on the total size. In one case (i.e., Patient 3), the tumor elicited the stenosis of the pharyngeal tube and dislocation of the base of the tongue anteromedially. However, the lesion occasionally induced the obstruction of left eustachian and Rosenmuller’s fossa (Patient 4). Lesions typically showed capsular calcification and uneven contrast agent reception. 

**Fig 1:**
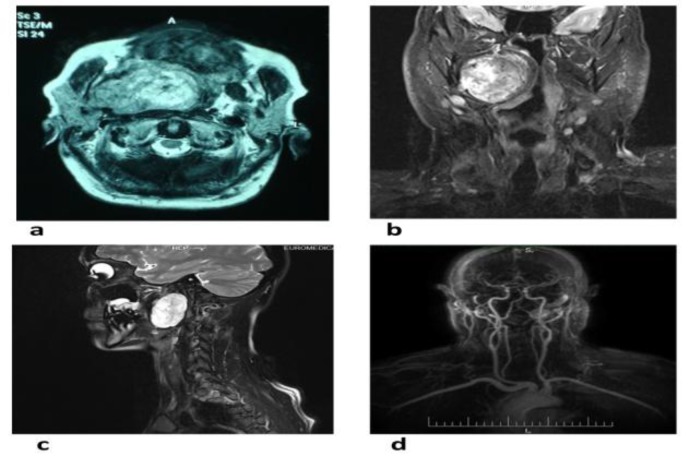
Computed tomography and magnetic resonance imaging scans in axial (a), coronal (b), and sagittal (c) planes demonstrating giant parapharyngeal space tumors lesions laid in the prestyloid compartment, (d) implementation of magnetic resonance angiography (Patient 6) due to superomedial tumor localization that raised concern about its relationship to adjacent vital vascular structures

A contrast-enhanced magnetic resonance imaging (MRI) scan verified the presence of a pear-shaped mass in the PPS and confirmed its dimensions. Tumors typically appeared with a low signal intensity in T1 and a low or nonhomogenous signal in T2-weighted sequence (Fig.1). On the other hand, magnetic resonance angiography was not considered a crucial prerequisite and was performed only once (on Patient 6) in our series due to the superomedial tumor localization, which raised concern about its relationship with the adjacent vital vascular structures (Fig. 1d).

Imaging investigations indicated that all but one of the lesions were over 4 cm. In this regard, the largest lesion measured 7.5 cm in its greater dimension. All lesions were in the prestyloid compartment and had dislocated the ICA posterolaterally. Such a tumor localization is of significant importance in selecting the proper surgical approach. In most of our cases, a combination of imaging modalities already revealed the benign nature of the lesions located in the prestyloid PPS. However, a preoperative fine needle aspiration biopsy (FNAB) was typically performed to confirm the tumor pathology. 

Table 1 summarizes the patients’ demographic data, clinical findings, and tumor size. The protocol of the investigation was approved by the Aristotle University of Thessaloniki in Greece. In line with the research ethical principles, written informed consent was obtained from each participant.

**Table 1:** Demographic data, clinical findings, tumor size, and surgical outcomes of the patients

Patients	Gender	Age	Symptomatology / clinical examination	Histological diagnosis	Tumor size (cm)^1^	Hospital stay (days)	Complications	Recurrence	Follow-up (months)
Patient 1	M	46	-Remittent headaches -Oral cavity foreign body sensation -Episodes of sleep apnea -Soft palate bulging -Ipsilateral tonsil dislocation	Pleomorphic adenoma	6.4×3.2×3.3	2	No	No	72
Patient 2	F	62	-Oral cavity foreign body sensation -Episodes of sleep apnea -Stertorous sleep -Soft palate bulging	Pleomorphic adenoma	6.2×5.5×5.8	2	No	No	56
Patient 3	F	67	-Dysphagia -Mild remittent dyspnea -Soft palate bulging -Ipsilateral tonsil dislocation	Pleomorphic adenoma	4.5×3.5×7.5	2	Mild postoperative pain	No	32
Patient 4	F	32	-Oral cavity foreign body sensation -Ipsilateral otalgia -Dysphagia -Lateral lingual swelling -Soft palate bulging -Ipsilateral middle ear effusion	Pleomorphic adenoma	4.9×4.3×5	2	No	No	20
Patient 5	F	72	-Dysphagia -Soft palate bulging	Pleomorphic adenoma	5.8×4.3×5.5	3	No	No	14
Patient 6	M	42	-Asymptomatic -Mild soft palate bulging	Pleomorphic adenoma	2.9×2.6×1.8^2^	2	No	No	10
Patient 7	M	47	-Asymptomatic -Soft palate bulging	Pleomorphic adenoma	5.1×3×5.4	2	Mild postoperative pain	No	8

M: Male, F: Female, 1: measured by imaging modalities, 2: superomedial position

## Results

As surgical excision is the mainstay of PPS tumor treatment, all of our cases were managed in a uniform way. Accordingly, surgical excision was recommended to all patients, and their informed consent was obtained. An additional consent was also obtained for conversion to an approach involving a possible mandibulotomy in case of the inadequacy of transoral exposure or observation of a malignant lesion. Intravenous antibiotics, most commonly cefuroxime, and intravenous dexamethasone were administered on call to the operating room. 

As far as the surgical technique is concerned, excision via a transoral approach, combined with a secondary trancervical approach, was designated as the optimal treatment. After orotracheal intubation contralateral to the neoplasm, the procedure was commenced with a transcervical approach under general anesthesia. A transverse, curvilinear incision was fashioned in a skin crease, two finger-breadths below and behind the angle and ramus of the mandible. This incision was designed to permit conversion to a more extensive approach, in case of encountering unanticipated findings, such as malignancy necessitating lymphadenectomy. 

Although FNAB had confirmed the benign nature of the lesions, the patients were subjected to intraoperative frozen section biopsy. Subplatysmal flaps were elevated, and the great auricular nerve was isolated and preserved. Both submandibular gland and marginal mandibular nerve were identified and preserved by being reflected superiorly and anteriorly. Removal of the submandibular gland was unnecessary since the rational of our secondary transcervical approach was the identification of vital structures rather than tumor excision. Accordingly, the maximal exposure of PPS was not a primary goal. The hypoglossal nerve was always identified and properly protected. Superficial cervical fascia was incised parallel to the ventral margin of the sternocleidomastoid muscle, which was identified and used to find the digastric muscle and the adjacent stylohyoid muscle, which were sequentially retracted.

**Fig 2:**
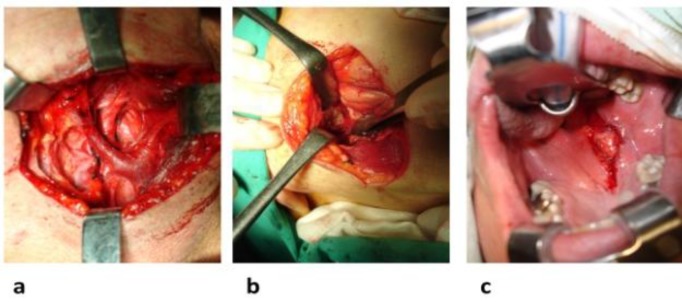
(a) Identification and preservation of the trunk of the jugular vein, common carotid artery, vagus nerve, and facial, lingual, and superior thyroid arteries, (b) confrontation with the posterior boundaries of the tumor capsule, and (c) incision over the bulging tumor giving access to the lateral wall of the parapharyngeal space

In the same stage, the trunk of the jugular vein and the common carotid artery were identified and secured. Finally, the vagus nerve and the facial, lingual, and superior thyroid arteries were identified and preserved (Fig.2a), which well delimited the operating field and facilitated the exposure and recognition of the posterior boundaries of the tumor capsule (Fig.2b). 

At this stage, our “secondary” transcervical approach was completed, and no effort was undertaken to manipulate or dissect the tumor through that approach since the mandible barrier would constitute a serious drawback. Similarly, no division of the stylohyoid ligament, stylohyoid muscle, or posterior belly of the digastric branch of the facial nerve was considered. The procedure continued with the “primary” transoral approach. To this end, a Boyle-Davis mouth gag was inserted to retract the tongue and facilitate ample oral cavity visualization. The retractor was released every 20 min to help alleviate the tongue swelling. An incision was fashioned over the bulging tumor to extend from the upper part of the soft palate passing parallel and medial to the pterygomandibular raphe, giving access to the lateral wall of the PPS (Fig. 2c). 

**Fig 3:**
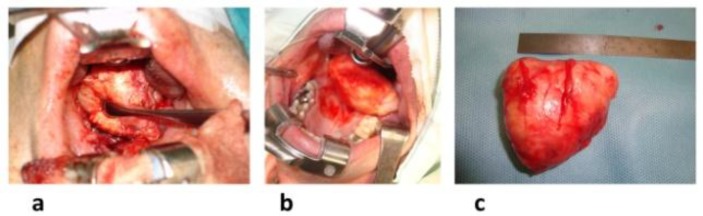
(a) Careful transoral tissue-conserving dissection between the tumor capsule and the surrounding tissues by gently dividing the soft tissue and fibrous adhesions with blunt instrument, (b) mobilization and excision of tumor in its entirety preventing spillage, and (c) analysis of excised specimen to ensure capsule integrity

It was avoided to make a far lateral incision to the soft palate as far as possible in order not to damage the palatine artery and neurovascular bundle. The incision was carried out through the mucosa, submucosa, and eventually the musculature of the anterior tonsillar pillar and muscle fibers of the superior pharyngeal constrictor muscle to find the fatty plane within the PPS. Once the muscular plane was dissected, the anterior aspect of the encapsulated tumor emerged and was visualized with the aid of a head light. Simultaneously, the “secondary” transcervical approach offered an excellent control of the vital structures, such as the carotid artery, internal jugular vein, and lower cranial nerves. Careful transoral tissue-conserving dissection was performed between the tumor capsule and surrounding tissues by gently dividing the soft tissue and fibrous adhesions via a blunt instrument or finger dissection (Fig.3a). Tumor could then be mobilized and excised in its entirety to avoid spillage (Fig.3b). The excised specimen was removed transorally and analyzed to ensure the capsule integrity (Fig.3c). 

**Fig 4:**
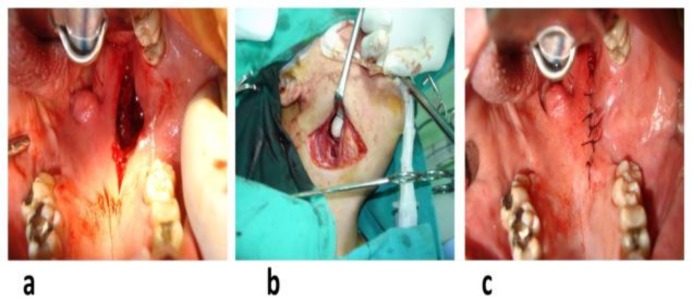
(a, b) Copious irrigation of the wound with saline solution and securing absolute hemostasis upon the completion of tumor removal and (c) closure of oral cavity incision in layers

Upon the completion of tumor removal, the wound was copiously irrigated with saline solution, and absolute hemostasis was secured prior to closure (Fig.4a,b). The oral cavity incision was closed in layers with absorbable sutures placed in the muscle plane, as well as submucosal tissues and mucosa. It was ensured that various muscle layers were accurately reapproximated to prevent velopharyngeal dysfunction. Watertight closure is essential for the prevention of salivary tissue erosion (Fig.4c). The external wound resulting from the “secondary” transcervical approach was also closed in layers following suction drain insertion.

Mean operating time was 76 min (skin incision to closure; range: 63-130 min) in the patients. Postoperatively, all patients received a short precautionary antibiotic treatment (i.e., cefuroxime) for a two-day period, as well as a short-term postoperative steroid therapy (i.e., single-dose intravenous dexamethasone). Nasogastric tube insertion was not considered an option, and all patients were put on a liquid diet on the first postoperative day and limited to a soft diet for 10 days. Oral cavity foreign body sensation as the most common symptom was resolved completely in the first postoperative period. All patients enjoyed an uneventful hospital stay and were discharged after a mean period of 2.1 days (with a maximum of 3 days).

Patients’ follow-up included clinical examination and a periodical annual MRI. The sessions were planned 1, 3, and 6 months after the first year of the surgery and repeated annually thereafter. The patients were followed up in our series for a maximum of 6 years, whereas the last patient was operated 8 months ago. The follow-up revealed no postoperative complications or signs of tumor recurrence in any of the patients.

## Discussion

The PPS, found lateral to the pharynx, is an inverted pyramid-shaped space with its apex starting at the greater cornu of the hyoid bone and its base ending at the base of the cranium, bearing heterogeneous content. It is divided into prestyloid and poststyloid spaces by the tensor-vascular-styloid fascia (7,8).

Determination of the tumor location is crucial in determining its benign or malignant nature and choosing the surgical technique for approaching and excising the tumor with minimal morbidity. 

The origin, location, and dimensions of PPS masses are assessed by imaging studies, which facilitate the separate recognition of prestyloid and poststyloid tumors. Sufficient PPS imaging can be achieved with contrast-enhanced CT and MRI modalities (9). In our series, a combination of imaging modalities assisted the diagnosis of a benign encapsulated tumor located in the prestyloid PPS. 

The suitability of using preoperative FNAB for PPS tumors has been debated in the international literature. Although the usefulness of this biopsy procedure in preoperative treatment planning is not under discussion, the sensitivity of this method has remained unacceptable and poor. However, in the present study, all patients were subjected to a preoperative FNAB in order to further affirm the imaging conclusions. Therefore, the diagnosis of a pleomorphic adenoma was preoperatively established via FNAB and intraoperatively confirmed with frozen section biopsy.Anatomic complexity of the PPS, in addition to its deep location and surrounding vital structures, makes the resection of the tumors located within this space a challenging measure. A plethora of surgical procedures have been utilized in the management of these lesions (10), including transoral, transcervical, transparotid, modified transcervical (i.e., transcervical-transparotid and transcervical-transmastoid), transmandibular, combined transcervical-transpharyngeal, and various craniofacial approaches (e.g., orbitozygomatic–middle fossa, infratemporal, and lateral skull base approaches) (6). 

An optimal surgical approach by definition should be the safest and most effective method facilitating a complete tumor removal and preventing injury to the vessels and nerves of the upper neck. Intraoperative visibility of all adjacent structures is an issue of paramount importance in performing a safe and radical dissection of the lesion. On the other hand, the approach should be implemented as conservatively as possible in order to provoke minimal functional and cosmetic side effects. This is a critical prerequisite for achieving an uncomplicated postoperative course and ensuring patient wellbeing. 

Although a detailed consensus on the principles governing the selection of the surgical approach is not currently available, there are certain tumor characteristics that are considered as the main parameters when deciding on optimal PPS surgical approaches. In this respect, the overall size of the PPS lesion, its location regarding vital vascular structures, and its malignant potential have long dictated the selection of the surgical treatment (11). 

According to the above principles, some extravagant approaches are reserved for the tumors with special characteristics. For example, craniofacial approaches are reserved for particularly large PPS tumors involving the temporal bone and skull base or extending into the infratemporal fossa, clivus, petrous bone, or nasopharynx (12,13). 

Regarding this, it is safe to argue that three approaches constitute the rule for dealing with most of the PPS tumors. These approaches include the transcervical (with or without a mandibulotomy), transoral, and less commonly transparotid approaches.

The transcervical approach, first described in 1955 (14), is considered the optimal and most frequently used method for the resection of the majority of PPS tumors (10,15-17). Many researchers have successfully used the transcervical approach for the resection of the tumors of the PPS in 90-100% of their patients (18,19). Yet, others suggest that although this approach is suitable for the removal of the small lesions confined in the lower PPS (20), it is inadequate for dealing with larger tumors. Therefore, it is argued that a maximum diameter of 4 cm roughly constitutes the limit beyond which the mandible poses a serious obstacle regarding tumor manipulation and safe excision (13,15,21). 

In our series, all lesions were greater than 4 cm, except for the case of ‘Patient 6’, who was subjected to an alternative approach due to the superomedial position of the lesion rather than its overall size. Many solutions have been suggested to overcome the mandible obstacle and achieve a wider PPS exposure. These solutions can be roughly divided into two distinct groups, including the retraction of the mandible in a protruded position and different mandibulotomy techniques (6,22,23). 

Mandibulotomy or mandibulectomy may be used in conjunction with the external approaches and has been recommended for large neoplasms, malignancies, vascular tumors, and occasionally lesions that require the interposition grafting of the internal carotid artery (2,24). One should bear in mind that adequate tumor visualization is a crucial goal, which facilitates en bloc tumor removal, achievement of hemostasis, and preservation of the adjacent nerves and vessels. Removal of large lesions, without the mandible division, remains an overall blind procedure that increases the probability of tumor rupture and spillage in an inaccessible area.

Larger tumors can be alternatively excised via a transparotid approach. Such a technique is used for deep lobe parotid tumors to facilitate facial nerve preservation (6). Combination of the above approaches would result in the transparotid-transcervical approach by extending the cervical incision superiorly, as for a parotidectomy. The main disadvantage of this approach is the probability of inducing facial nerve injuries during surgical handling, which can lead to postoperative neurapraxia or even the paralysis of the nerve. 

Furthermore, some authors argue that a lesion with a maximum diameter of 4 cm can be also managed through this approach. However, they also believe that when tumor adhesion is dense (e.g., in patients who have previously undergone biopsy attempts), the transparotid approach bears a significant risk of complications (21).

 The transoral approach described by Ehrlich in 1950 is the most controversial PPS surgical approach (25). Works published by McElroth et al. in 1963 describe the use of this approach (26), along with the external carotid artery ligation to remove PPS lesions in 112 patients. Later, in 1988, Goodwin and Chandler reported their experience with the transoral approach for the resection of the small tumors of the PPS (27), which appeared avascular on imaging studies. They reported that this approach gave both adequate and direct access to PPS for the patients presenting with tumors fulfilling the above criteria. 

Yet, as the literature suggests, most of the researchers disaffirmed these beliefs and expressed caution that the transoral approach has low credibility and should be altogether avoided (1,2,10,11,28). 

The risk of hemorrhage, cranial nerve injury, tumor spillage/implantation, and inhibited access were the main factors explaining the reasons for the selection of transoral approach for a blind surgical exploration and its cautious adoption for selected cases (5,20). 

Critics of this approach stress that the risk of tumor rupture is extremely high; therefore, they discredit the selection of the procedure with the exception of particularly small (<2 cm) well-circumscribed tumors that are not palpable in the neck or parotid gland but project into the oropharynx (21,29). Nevertheless, to add to the overall controversy, O’Malley et al. recently revisited transoral approaches for PPS management with robotic assistance (30), reporting excellent tumor control rates with no increase in postoperative infections. Similarly, others have reported good results with the transoral excision of PPS tumors (31), even when dealing with the isolated cases of particularly sizeable lesions (32). 

In our series, all but one patients had large PPS lesions over 4 cm. After careful preoperative workup, it was decided to excise the tumors transorally in order to avoid approaches with greater morbidity, higher potential complication rate, and prolonged hospital stay. Yet, weighing up the fact that an exclusively transoral approach has been widely criticized concerning the safety of important adjacent neurovascular structures, we complemented the transoral excision with an accessory transcervical approach. Secondary approach was performed solely to necessitate optimal neurovascular control rather than tumor manipulation.

As a rule, tumor accessibility, radicality of resection, and overall safety are the prime considerations in selecting a surgical approach. When efficacy and safety are established, good judgment suggests the selection of the approach that results in minimum functional morbidity and aesthetic deformity. In this context, our transoral approach provided excellent tumor manipulation for prestyloid lesions, while the secondary transcervical approach effectively identified and secured the critical structures, though minimally concerning the dissection of the tumor itself. 

The results showed that there was no intraoperative tumor spillage and recurrence after a maximum follow-up period of 6 years, suggesting the improved efficacy of this approach. Furthermore, the mean operative time was kept short, compared to that of the more extensive approaches, whereas the duration of postoperative hospital stay was minimal. In the current study, the researchers believe that transoral excision brings the neoplasm directly into view in the superficial portion of the dissection. As a result, it is advantageous to achieve the visual control of dissection over the medial side and upper parts of the tumor and prevent blind blunt dissection usually required when selecting an external approach.

 Yet, the avoidance of serious potential complications cited for alternative surgical techniques seems to be the distinct and major advantage of the selected procedure. A transcervical approach does not afford adequate exposure because the superior extent of the tumor is usually quite distant from the cervical incision. In such cases, the capsular rupture and spillage of an encapsulated tumor during the removal of a large bulk through a limited space carries a significant risk (33). 

Furthermore, our experience demonstrated that the transoral excision of PPS tumors may offer a decrease in the risk of overall complications when compared to the published reports of the more commonly used transcervical approach having complication rates as high as 30-40% (10,16,19). Additionally, since the presence of the mandibular ramus prevents direct access to this region when dealing with the lesions of great volume (>4cm), the transcervical approach is often complemented with at least an anterior subluxation of the mandible or more frequently a mandibulotomy. 

Both of these procedures result in significant postoperative morbidity and potential complications. Therefore, the excessive distraction of the mandible or incision into the temporomandibular joint capsule commonly leads to permanent damage to the articular meniscus or ligaments, prolonged immobilization or dysfunction of these structures, and even trismus often resistant to treatment (34). 

On the other hand, mandibulotomy can result in a series of postoperative complications depending on the type of osteotomy performed. Such complications include malocclusion, nonunion, loss of dentition, iatrogenic paralysis of the mandibular branch of the facial nerve, and planned inferior alveolar nerve section, which results in the hypaesthesia of the inferior lip and mandible (36). Osteotomies placed at the angle of the mandible have even been reported to cause damage to the lingual nerve (35). More extensive approaches, such as midline mandibulotomy, frequently demand a temporary tracheostomy and almost always prolonged nasogastric intubation (20,34), which both demand prolonged hospitalization raising to an average of 10-14 days (6). 

Moreover, such approaches necessitate an inferior lip-splitting incision leading to a visible scar and aesthetic deformity. On the contrary, none of the patients treated in the current study experienced any complications or serious postoperative morbidity. In our patients, hospitalization was confined to a maximum of 3 days, depicting a better overall postoperative course. In addition, all patients appreciated the absence of lip and mandible splitting and the small externally visible cervical scar, easily camouflaged with skin crease. All patients started oral intake on the first day postoperation, and none of them showed any respiratory complications necessitating a tracheostomy. Finally, transparotid approach has limited applicability on deep-lobe parotid tumors and involves the manipulation of the facial nerve; therefore, its potential palsy is often anticipated. A salivary fistula (20,36) can be also considered as a potential complication. Despite our results, it is important to note that a transoral excision is not required in every case of a PPS tumor. It goes without saying that the lesions having appropriate volume and location for excision via a sole transcervical approach should not be dealt with a transoral approach since such a redundant procedure has nothing extra to offer. 

Furthermore, when transoral approach is considered as the main treatment, it is imminent to determine preoperatively the characteristics of the tumor (i.e., being benign and well encapsulated), while a hypervascular or dumbbell-shaped or even a possibly malignant tumor can be ruled out with the aid of CT or MRI examination. It is imperative to note that as depicted by imaging modalities, all lesions laid in the prestyloid compartment dislocating the ICA posterolaterally. 

This fact was of crucial importance in adopting the appropriate surgical approach. Consequently, a careful preoperative diagnostic procedure that must take advantage of imaging studies (e.g., CT and MRI) and cytology (FNAC) is of paramount importance in order to plan surgical treatment with a safe approach, ensuring reduced complication rate, aesthetic-functional damage, and recurrence.

Large tumor size was the main reason of selecting the transoral excision in our series. Yet, ‘Patient 6’ was a separate case for the safe excision of which transcervical approach was inadequate despite having a lesion of small size (<4 cm) because the mentioned method provided minimal visualization and manipulation of the tumor. Superomedial position of the lesion dictated a transoral excision because this surgical technique provided sufficient exposure for en bloc resection, which is superior to the one achieved by more extensive approaches, especially at the far limit of the exposure. It has been also stated in the literature that transoral approach can be advantageous when surgically accessing the superomedial PPS (20).

## Conclusion

In the present study, transoral approach was proposed as an efficient way to excise the benign well-defined tumors of the prestyloid PPS. This case series indicated that this approach can be adopted for the PPS masses of great volume, provided that they fulfill the above criteria. This procedure appears to be safe if applying a minimal secondary auxiliary transcervical approach. This approach is associated with lower morbidity, fewer complications, and shorter hospital stay since it does not require the implementation of more extensive approaches, especially mandibulotomy. 
